# Genome-Wide Analysis of the Polyphenol Oxidase Gene Family in *Olea europaea* Provides Insights into the Mechanism of Enzymatic Browning in Olive Fruit

**DOI:** 10.3390/antiox12091661

**Published:** 2023-08-23

**Authors:** Qingqing Liu, Chenhe Wang, Qizhen Cui, Yutong Fan, Jianguo Zhang, Guodong Rao

**Affiliations:** 1 State Key Laboratory of Tree Genetics and Breeding, Research Institute of Forestry, Chinese Academy of Forestry, Beijing 100091, China; liuqingqing2000@126.com (Q.L.);; 2 Collaborative Innovation Center of Sustainable Forestry in Southern China, Nanjing Forestry University, Nanjing 210037, China; 3 Key Laboratory of Tree Breeding and Cultivation, National Forestry and Grassland Administration, Research Institute of Forestry, Chinese Academy of Forestry, Beijing 100091, China

**Keywords:** *Olea europaea*, fruit browning, polyphenol oxidase, monophenolase activity, diphenolase activity, gene expression

## Abstract

Browning of olive (*Olea europaea* L.) fruit reduces the sensory and nutritional qualities of olive oil, thereby increasing production costs. Polyphenol oxidases (PPOs) are the key enzymes that catalyze phenolic substance oxidation and mediate enzymatic browning in olive fruit, but the exact regulatory mechanism remains unclear. The main challenge is the lack of comprehensive information on OePPOs at the genome-wide level. In this study, 18 *OePPO* genes were identified. Subsequently, we performed a bioinformatic analysis on them. We also analyzed the expression patterns and determined the relationship among browning degree, PPO activity, and expression of *OePPOs* in the fruits of three olive varieties. Based on our analysis, we identified the four most conserved motifs. OePPOs were classified into two groups, with OePPOs from Group 1 showing only diphenolase activity and OePPOs from Group 2 exhibiting both mono-/diphenolase activities. Seven pairs of gene duplication events were identified, and purifying selection was found to have played a critical role in the evolution of the OePPO gene family. A positive correlation was observed between the browning degree of olive fruit and PPO activity across different olive varieties. Moreover, two important genes were found: *OePPO-5* the main effector gene responsible for fruit browning, and *OePPO-8*, a key gene associated with specialized metabolite synthesis in the olive fruit. In short, our discoveries provide a basis for additional functional studies on *OePPO* genes and can help elucidate the mechanism of enzymatic browning in olive fruit in the future.

## 1. Introduction

The olive plant (*Olea europaea* L.) is an oil crop belonging to the Oleaceae family and Olea genus. The extraction of olive oil from olive fruit results in a product that is rich in nutrients and bioactive compounds [1], earning it the reputation of being the “Queen of Plant Oils”. As a result, the olive has become an important economic species [2].

Polyphenol oxidases (PPOs) are a type of metalloenzyme that contains a type III copper center. They are widely found in archaea, bacteria, fungi, plants, and animals [3,4]. It is widely accepted that the PPO family consists of three different types of enzymes: tyrosinases (TYRs) [5], catechol oxidases (COs) [6], and aurone synthases (AUSs) [7]. TYRs are bifunctional PPOs that possess the ability to catalyze the ortho-hydroxylation of monophenols to diphenols (monophenolase activity, EC 1.14.18.1), as well as the oxidation of diphenols to form o-quinones (diphenolase activity, EC 1.10.3.1), while COs only possess the diphenolase activity [8,9]. AUSs were initially believed to represent a novel class of PPOs and were found to play a significant role in the formation of chalcone-derived compounds known as aurones [10]. However, subsequent research reclassified AUSs as a type of catechol oxidases (COs) [11]. TYRs and COs were once considered indistinguishable based on their amino acid sequences [12,13], whereas recent studies have provided valuable insights into the functional differentiation within the PPO enzyme family. The researchers used the tyrosinase domain sequences of plant PPOs that were clearly biochemically characterized for phylogenetic analysis, successfully differentiating TYRs with monophenolase activity and COs with only diphenolase activity into two distinct groups [14].

In plants, PPOs are typically expressed in an inactive form as proenzymes, with a molecular weight of 54–62 kDa. This proenzyme form of PPOs includes an N-terminal transit peptide of approximately 8–12 kDa and a C-terminal domain of around 15–20 kDa [15]. The presence of the C-terminal domain is believed to hinder the activity of PPOs when the C-terminal domain undergoes proteolytic cleavage, enabling PPOs to initiate their catalytic function [16]. Apart from the N-terminal transit peptide and C-terminal domain, the core sequence of PPOs contains conserved CuA and CuB domains (tyrosinase domain, Pfam00264). The CuA and CuB domains each contain three conserved histidine residues, which are utilized to bind copper ions [11]. Relevant research has shown that the sequence of CuA and CuB domains determines whether a PPO has monophenolase activity [11,17].

Browning is a common phenomenon in plants, and PPOs are well-known for their involvement in the browning reaction. In normally growing plants, PPOs are typically localized in chloroplasts [18,19]. PPOs could catalyze phenolic compounds which are localized in vacuoles such as catechol, caffeic acid, chlorogenic acid, protocatechuic acid, gallic acid, and others [20]. During post-harvest processing and storage, various factors such as aging, damage, and interactions with pests and pathogens can lead to contact between PPOs and phenolic substrates [19,21]. Then, under the presence of oxygen, PPOs catalyze the phenolic substrates to produce o-quinones. Quinones undergo spontaneous polymerization and combine with amino acid residues in protein side chains to produce black or brown substances (melanoidins) [22,23,24], resulting in browning [25]. This process has a negative impact on the color, flavor, nutritional properties, and shelf life of food products [26]. However, in some cases, enzymatic browning may be beneficial as it leads to the formation of compounds that impart characteristic flavors and aromas [27]. In addition, PPOs also play an important role in the synthesis of plant metabolites. In the acteoside biosynthesis pathway, PPOs catalyze the conversion of tyrosine, tyramine, and tyrosol into their corresponding diphenolic products, DOPA, dopamine, and hydroxytyrosol [28]. In olives, PPOs are also speculated to be involved in the synthesis of characteristic metabolites such as hydroxytyrosol and oleuropein [29].

In this study, OePPOs were identified from the entire olive genome, and their gene structures, phylogenetic relationships, chromosomal locations, and collinearity were analyzed. Furthermore, the expression patterns of these PPOs in different tissues of the olive plant were investigated. Herein, we aimed to predict the types (TYRs or COs) of the 18 identified olive PPOs (OePPOs), determine the relationship between browning degree and PPO activity in different olive fruit varieties, and identify the main effector genes involved in browning within the OePPO family.

## 2. Materials and Methods

### 2.1. Identification and Physicochemical Properties of OePPO Genes

The high-quality olive genome (CRA003087) used to identify the *PPO* genes was obtained using Oxford Nanopore third-generation sequencing and Hi-C technology by our laboratory team and has been submitted to the China National Center for Bioinformation (CNCB, https://bigd.big.ac.cn/, accessed on 1 February 2023) [29]. A local blastp search was first run with default parameters utilizing known eggplant PPOs sequences [30] and three poplar PPOs sequences (PtdPPO1, 2, 3, GenBank accessions AF263611, AY665681 and AY665682) [31] as the query. Secondly, a Hidden Markov Model (HMM) was constructed using the PPO sequences of eggplant and poplar, and the HMMER3.0 (http://hmmer.org/, accessed on 1 February 2023) tool was employed to search for PPO protein sequences in olives. The sequences obtained from the two methods were merged, following which all candidate sequences were submitted to an NCBI Batch CD-search (https://www.ncbi.nlm.nih.gov/Structure/bwrpsb/bwrpsb.cgi, accessed on 1 February 2023) to identify their conserved domains. After manual screening, 18 sequences were considered to be PPO family members.

The online tools TMHMM (https://services.healthtech.dtu.dk/services/TMHMM-2.0/, accessed on 1 February 2023) and ExPASy (https://web.expasy.org/compute_pi/, accessed on 1 February 2023) were used to calculate the transmembrane domains (TMHs), theoretical isoelectric points (Pis), and molecular weights (MWs) of the olive PPO proteins.

### 2.2. Phylogenetic Analysis, Motif Pattern, and Gene Structure Analysis of OePPO Genes

Multiple sequence alignment was conducted among the amino acid sequences of all PPO proteins in *Olea europaea* and some representative PPO proteins that are known to be either TYRs or COs (Table S3 [14]) with ClustalW using the default parameters in MEGA7.0 software [32]. The neighbor-joining method was used to establish a phylogenetic tree based on this alignment with 1000 bootstrap replicates. The evolutionary distances were obtained using the p-distance method and iTOL was used to modify the phylogenetic tree [33].

At the same time, the protein data for *Populus trichocarpa*, *Solanum lycopersicum*, *Sorghum bicolor*, *Selaginella moellendorffii*, and *Physcomitrium patens* were downloaded from NCBI (GCF_000002775.4, GCF_000188115.4, GCF_000003195.3, GCF_000143415.4, and GCF_000002425.4). Through blastp and annotation information, we identified their polyphenol oxidase families. Subsequently, comprehensive comparisons of these PPOs and OePPOs were performed.

The amino acid sequences of the OePPOs were analyzed by the MEME program to identify conservative motifs [34]. The gff information of the OePPOs was then used to predict the exon–intron organizations through the online gene structure display service 2.0 [35].

### 2.3. Prediction of Cis-Acting Elements

The upstream 2000-bp regions of OePPOs were submitted to PlantCARE to identify cis-acting elements [36].

### 2.4. Analysis of Chromosome Mapping and Duplication Events

The location information of the genes on the chromosome was extracted from the gff file using Tbtools [37]. Tandem duplication and segmental duplication events were analyzed by the one-step McscanX program in Tbtools [37].

### 2.5. Expression Patterns of OePPOs

To explore the expression patterns of OePPOs in different tissues, the original RNA-seq data in fastq format were obtained from the ebi-ena database (https://www.ebi.ac.uk/ena/browser/home, accessed on 1 February 2023), with the accession numbers PRJNA590386 (roots, stems, leaves, flowers, fruits, and meristems) and PRJNA596876 (fruits, new leaves and old leaves). Our laboratory team analyzed the data [38,39,40,41,42,43], and the expression levels of the genes were normalized by fragments per kilobase of transcript per million mapped reads (FPKM). We then calculated the FPKM values of OePPOs and conducted an analysis.

### 2.6. Browning Degree and PPO Activity Assay of Three Olive Varieties

Fruits were sampled from three dwarf and closely planted olive varieties, namely Arbequina (F1), Arbosana (F2), and Koroneiki (F3). The fruit samples were added to pre-cooled phosphate buffer (0.1M, pH 7.0) in a ratio of 1:10 (w:w). The samples were then ground using a micro ball mill GT3000 (Powteq, Beijing, China) at 1500 rpm for 1 min. Then, the samples were centrifuged at 4 °C and 12,000 rpm for 15 min. The supernatant was collected and used as the PPO enzyme source. 

Browning degree assay: The supernatant was incubated in a water bath at 37 °C for 15 min. After incubation, the absorbance (A) was measured at 416 nm wavelength. The value of A_416_ represents the browning degree.

Enzyme activity assay: The enzyme activity assay system consisted of 1 mL, including 800 μL phosphate buffer, 100 μL 0.1 mol/L catechol, and 100 μL enzyme source. The absorbance was recorded at 400 nm every 30 s for a total of 3 min. Under the measurement conditions, a change of 0.01 in A400 per minute per gram of sample represents one unit of polyphenol oxidase activity {U/(g·min)}.

Excel 2021 was used to perform the statistical analysis for all the data, calculate the standard deviation, and create graphs. The significance analysis was conducted using SPSS 26.

## 3. Results

### 3.1. Identification and Physicochemical Properties of OePPOs

A total of 18 *OePPOs* were selected through local blastp and screened based on the presence of the tyrosinase domain structure (Table S1). They were named *OePPO-1* to *OePPO-18*. Subsequently, their physicochemical properties were analyzed and presented in (Table S1). Previous studies [31,44,45] have indicated that plant PPOs typically possess three typical conserved domains, the tyrosinase domain, PPO_DWL domain, and PPO_KFDV domain. Among these domains, the tyrosinase domain has been confirmed to be the catalytic and active domain of plant PPOs [11,17]. The 18 selected OePPOs all contain the tyrosinase domain, and 15 of them have a pfam accession number of pfam00264. OePPO-15, 16, and 18 have a pfam accession number of cl02830 instead of pfam00264. It is worth noting that pfam00264 belongs to the cl02830 tyrosinase superfamily. A total of 17 OePPOs possess the PPO1_DWL domain. OePPO-18 contains the PPO1_DWL domain belonging to the cl13563 superfamily. OePPO-1 to OePPO-14, OePPO-16, and OePPO-17 possess the pfam12142 domain, which is a part of the cl13563 superfamily. In total, 16 OePPOs possess the PPO1_ KFDV domain. OePPO-8, 18 contains the PPO1_ KFDV domain belonging to the cl15965 superfamily. OePPO-1 to OePPO-7 and OePPO-9 to OePPO-15 possess the pfam12143 domain, which is a part of the cl15965 superfamily.

The amino acid lengths of the protein family members range from 306 to 597 aa, the molecular weight varies from 41.6 to 66.94 kDa, and the isoelectric point ranges from 5.23 to 8.62. The transmembrane domain prediction results indicate that none of the OePPO protein family members contain transmembrane domains.

### 3.2. Evolution, Conserved Motifs, and Exon–Intron Organizations of OePPOs

**Figure 1:**
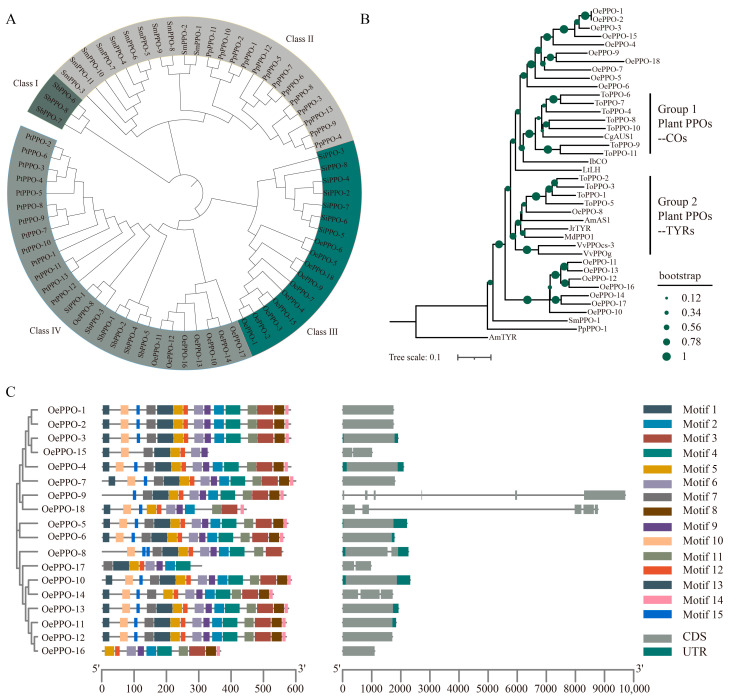
(**A**) Phylogenetic tree of plant PPOs; (**B**) phylogenetic tree constructed using TYR domain sequences of known naturally mono/dioxygenase-specific plant PPOs and OePPOs. The tree was rooted to a putative TYR sequence from the cyanobacterium *Acaryochloris marina* (GenBank: ABW32074.1), as well as to putative PPO sequences from the moss *Physcomitrella patens* (NCBI: XP_024397287.1), and the spike moss *Selaginella moellendorffii* (NCBI: XP_024519810.1); and (**C**) conserved motifs (on the left) and exon–intron organizations (on the right) of the OePPO gene family.

The phylogenetic tree was constructed using the amino acid sequences of 18 OePPOs, 13 *Populus trichocarpa* PPOs, 8 *Solanum lycopersicum* PPOs, 13 *Physcomitrium patens* PPOs, 11 *Selaginella moellendorffii* PPOs, and 8 *Sorghum bicolor* PPOs (Table S2). The results (Figure 1A) reveal that plant PPOs can be classified into four classes, with olive OePPOs distributed in Class III and Class IV. Some OePPOs (OePPO-1 to OePPO-7, OePPO-9, OePPO-15, and OePPO-18) belong to Class III, which also includes SiPPOs (PPOs from tomato) as they are both dicot plants. Other OePPOs (OePPO-8, 10-14, 16, and 17) are grouped in Class IV. In addition to OePPOs from the dicot plant olive, Class IV also includes PtPPOs from the dicot plant Populus and SbPPOs from the monocot plant sorghum. Meanwhile, SmPPOs from the fern plant *Selaginella moellendorffii* and PpPPOs from moss plant *Physcomitrella patens* are uniquely grouped in Class II, distinguishing them from the PPOs of other angiosperms. Tran and Taylor [31] mentioned that the PPOs in gymnosperms are not as widely expressed as the PPOs in angiosperms. The differences in phylogenetic classifications and expression patterns between angiosperms and gymnosperms may indicate that the PPOs in these two groups of plants have undergone distinct evolutionary events, suggesting the potential functional divergence of PPOs between them.

To further investigate the potential functional divergence of OePPOs, a phylogenetic tree was constructed by performing a systematic evolutionary analysis of the TYR domains of the 18 OePPOs along with the TYR domains of other plant PPOs whose activities have been previously studied. The species include *Antirrhinum majus*, *Coreopsis grandiflora*, *Juglans regia*, *Malus domestica*, *Tarraxacum officinale*, and *Vitis vinifera* [14] (Figure 1B). In Group 1, all the confirmed COs (catechol oxidases) of plant PPOs cluster together. In this group, ToPPO-4 and ToPPO-6 to ToPPO-11 of *Tarraxacum officinale* have been confirmed to have catalytic activity towards catechol (CAT, benzene-1,2-diol), 4-methylcatechol (4MC, 4-methylbenzene-1,2-diol), dihydroxyphe nylacetic acid (DOPAC, 2-(3,4-dihydroxyphenyl)acetic acid), dihy-droxyphenylalanine (L-DOPA, (S)-2-amino-3-(3,4-dihydroxyphenyl)propanoic acid), and dopamine (DA, 4-(2-aminoethyl)benzene-1,2-diol), but they do not have catalytic activity towards monophenols [46]. The PPOs mentioned (ToPPO-4 and ToPPO-6 to ToPPO-11) together with OePPO-1 to OePPO-7, OePPO-9, OePPO-15, and OePPO-18 from the Class III of olive PPOs form a larger branch, suggesting that the OePPOs in Class III may have CO functionality. It should be noted that while the OePPOs from Class III cluster together with other plant COs on the same larger branch, all OePPOs from Class III form a unique cluster on their own (Figure 1B). This suggests that these OePPOs may not only have CO functionality but also possess unique characteristics specific to olive.

In Group 2, there are the already identified TYRs from various plants. For example, JrTYR in this group has been demonstrated to have activity towards tyrosine [47]. The OePPOs from Class IV (OePPO-11, 16, 17, and 12-14) cluster together on the same larger branch, suggesting that Class IV OePPOs may have TYR functionality. Similar to Group 1, except for OePPO-8 which clusters together with other plant TYRs, other OePPOs from Class IV form a separate and independent branch (Figure 1B). Research has shown that TYR (+)-larreatricin hydroxylase (LtLH) serves as a boundary between COs and TYRs [14]. LtLH also divides OePPOs into two groups: OePPO-1 to 7 and OePPO-9, 15, and 18 cluster together with the COs of Group 1, while OePPO-8, 10-14, 16, and 17 cluster with the TYRs of Group 2.

To further investigate the OePPOs sequences, conserved motif analysis was performed, and 15 motifs were identified using MEME (Figure 1C, motif sequences could be found in Table S4). Among the OePPOs, OePPO-1 to OePPO-7 and OePPO-10 to OePPO-13 contain 15 motifs each; OePPO-8 contains 12 motifs, with motif 15 appearing twice; OePPO-9 contains 13 motifs; OePPO-14 contains 14 motifs; OePPO-15 contains 8 motifs; OePPO-16 contains 10 motifs; OePPO-17 contains 8 motifs; and OePPO-18 contains 11 motifs. Motifs 5, 6, 9, and 12 are the four conserved motifs present in all OePPOs. In OePPO-1 to OePPO-14 and OePPO-17, motifs 5, 6, 9, and 12 are completely present within the tyrosinase domain. In OePPO-15, 16, and 18, although motif 5, 6, 9, and 12 sequences are not entirely contained within their tyrosinase domain, they differ by at most five amino acids. Therefore, it can be inferred that these four motifs belong to the tyrosinase domain. Hence, these four motif sequences can be considered characteristic sequences for OePPOs.

Introns are acquired or lost during the process of evolution. Therefore, the counts and phases of introns provide insights into evolutionary relationships [48]. The exon–intron organizations of *OePPOs* (Figure 1C) show that *OePPO-9* and *OePPO-18* possess longer intron structures, containing five and three introns, respectively. Both of these genes belong to Class III of the *OePPOs*. *OePPO-8* and *OePPO-14*, belonging to Class IV of the *OePPOs*, were found to possess one shorter intron structure each. In addition to the four mentioned genes, the remaining fourteen genes were found to lack intron structures. This pattern has also been observed in other dicotyledonous plant *PPO* gene families, such as in the eggplant *PPO* gene family [45].

### 3.3. Prediction of a Cis-Acting Element

**Figure 2:**
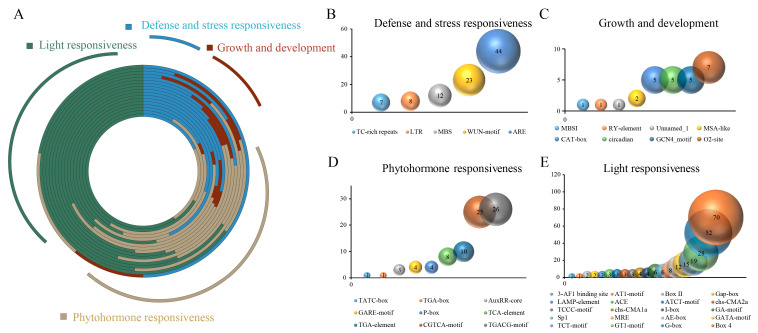
Cis-acting elements of OePPOs. (**A**) Circular plot showing the proportion of cis-acting elements in OePPO-1 to OePPO-18, from outer to inner circles; (**B**) defense and stress responsiveness elements; (**C**) growth and development elements; (**D**) phytohormone responsiveness elements; and (**E**) light responsiveness elements.

To understand the transcriptional regulation mechanisms, the PlantCARE database was used to analyze the cis-acting elements in the promoter regions of the *OePPOs*. Based on the functions of the cis-acting elements, they were classified into light responsiveness elements, phytohormone responsiveness elements, defense and stress responsiveness elements, and growth and development elements. As shown in Figure 2, among the 4 categories of elements, there are 8 types of growth and development elements, with the O2-site element appearing most frequently, 7 times (Figure 2C); 5 types of defense and stress responsiveness elements, with the ARE element appearing most frequently, 44 times (Figure 2B); 9 types of phytohormone responsiveness elements, with the TGACG-motif element appearing most frequently, 26 times (Figure 2D); and 20 types of light responsiveness elements, with the Box 4 element appearing most frequently, 70 times (Figure 2E). In the *OePPOs* family, the light responsiveness elements have the highest proportion, with an average occupancy of 48.29% per gene. Next are the phytohormone responsiveness elements and defense and stress responsiveness elements, with an average occupancy of 25.98% and 20.01% per gene, respectively. The number of growth and development elements is the lowest, with an average occupancy of 5.51% per gene (Figure 2A). The above results suggest that light may play a significant role in regulating the gene expression and activity of OePPOs. Previous research has shown that light stress can lead to PPO inactivation [49].

However, in the *OePPOs*, some genes appear to be more unique. For example, in *OePPO-8* and *OePPO-18*, the proportion of phytohormone responsiveness elements is the highest, accounting for 36.36% and 43.59%, respectively, which is higher than the second highest proportion of light responsiveness elements (31.82% and 38.46%, respectively). In contrast, in *OePPO-15* and *OePPO-17*, the proportion of defense and stress responsiveness elements is the highest, accounting for 47.35% and 50%, respectively, which is higher than the second highest proportion of light responsiveness elements (37.5% and 22.22%, respectively). This result suggests that *OePPO-15* and *OePPO-17* may play a major role in resisting environmental stress and adversity, similar to some previously reported plant PPOs [50].

### 3.4. Chromosome Locations and Duplication Events

**Figure 3:**
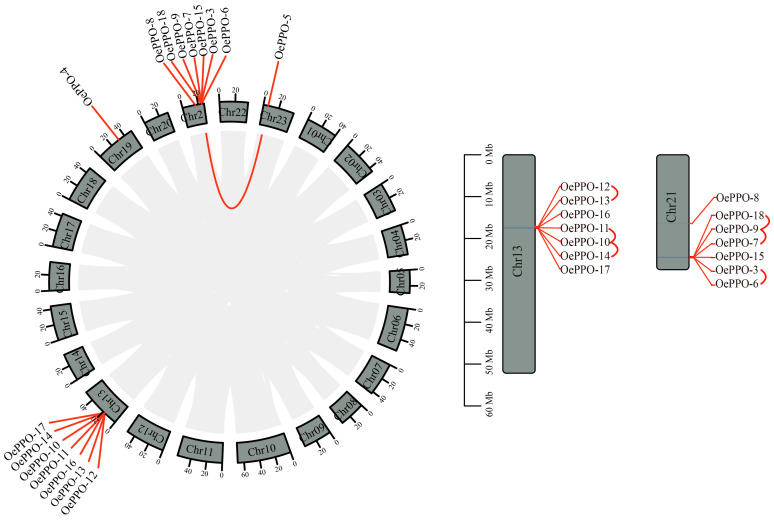
Chromosome locations and duplication events.

The chromosomal localization analysis was then performed according to the olive genome sequence file and annotation file. *OePPO-1* and *OePPO-2* are located on Contig01205 and Contig001268, respectively, while the rest of the *OePPO* genes are located on chromosomes (Figure 3). Inside the chromosomes, the distribution of *OePPOs* is not uniform: chromosome 13 and chromosome 21 have the highest density of *OePPOs*. For example, *OePPO-10* to *OePPO-14*, *OePPO-16*, and *17*, are all located on Chr 13, while *OePPO-3*, *OePPO-18*, and *OePPO-6* to *OePPO-9* are all located on Chr 21. Additionally, there is only one *OePPO* gene each on Chr 19 and Chr 23, which are *OePPO-4* and *OePPO-5*, respectively.

At the genome-wide level, we investigated the gene duplication events of *OePPOs*, which included six pairs of tandem events involving ten genes and one segmental event involving two genes: *OePPO-10* and *OePPO-11*, *OePPO-11* and *OePPO-14*, *OePPO-7* and *OePPO-9*, *OePPO-9* and *OePPO-18*, *OePPO-3* and *OePPO-6*, and *OePPO-12* and *OePPO-13*. These six pairs of genes form tandem repeat genes, showing close relationships and relatively short genetic distances. Additionally, *OePPO-5* and *OePPO-18* are segmental repeat genes, located on Chr23 and Chr13, respectively. It is noteworthy that *OePPO-5* and *OePPO-18* are involved in both tandem and segmental events.

Based on the above results, it can be observed that *OePPOs* might have expanded to their current size through both tandem and segmental duplication events, with tandem events likely being the major driving force. The selection pressure on gene pairs is commonly measured using the Ka/Ks ratio, which represents the ratio of the number of nonsynonymous substitutions per nonsynonymous site (Ka) to the number of synonymous substitutions per synonymous site (Ks). As shown in Table 1, the Ka/Ks ratios for *OePPOs* range from 0.15 to 0.58, with an average Ka/Ks value of 0.33. These results suggest that purifying selection may have played a critical role in the evolution of this gene family.

### 3.5. Expression Patterns of OePPOs in Different Olive Tissues

**Figure 4:**
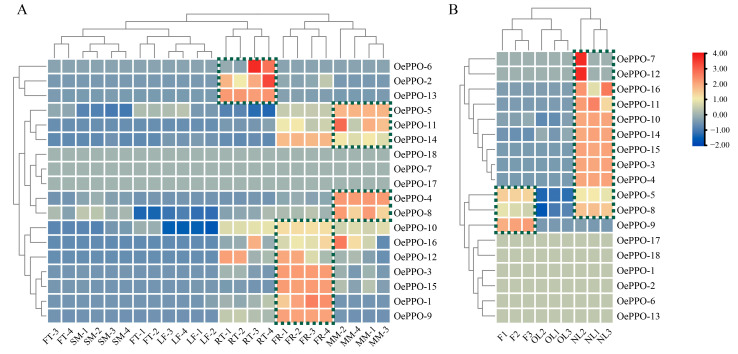
Expression patterns of the OePPOs in different tissues. (**A**) FT: fruit; SM: stem; LF: leaf; RT: root; FR: flower; and MM: meristem. (**B**) F: fruit; OL: old leaf; and NL: new leaf.

To gain further insight into the functions of the *OePPOs*, their RNA-seq expression patterns were quantified based on the FPKM values in six different tissues (fruits, flowers, leaves, meristems, roots, and stems) (Table S5, Figure 4A) and three different tissues (Table S6, Figure 4B). Figure 4A demonstrates that *OePPO-2*, *OePPO-6*, and *OePPO-13* are primarily expressed in roots, *OePPO-4*, *5*, *8*, *11*, and *OePPO-14* show predominant expression in flowers, and *OePPO-1*, *3*, *9*, *10*, *12*, *15*, and *OePPO-16* exhibit main expression in meristems. However, *OePPO-7*, *17*, and *OePPO-18* do not display detectable expression in any of these six organs. The expression patterns of *OePPO* genes vary in different organs, with predominant expression observed in the roots, flowers, and meristems. Interestingly, the genes primarily expressed in each tissue do not solely belong to Group 1 or Group 2 but are evenly distributed between the two groups. For instance, in the roots, *OePPO-2* and *OePPO-6*, both belonging to Group 1 and Class III, exhibit higher expression, while *OePPO-13*, which belongs to Group 2 and Class IV, also shows significant expression. In flowers, *OePPO-4* and *OePPO-5* from Group 1 and Class III are highly expressed, while *OePPO-8*, *OePPO-11*, and *OePPO-14* from Group 2 and Class IV also show notable expression. In the meristems, *OePPO-1*, *OePPO-3*, *OePPO-9*, and *OePPO-15* from Group 1 and Class III are predominantly expressed, while *OePPO-10*, *OePPO-12*, and *OePPO-16* from Group 2 and Class IV also exhibit significant expression. These results suggest that the OePPOs in olive may undergo functional differentiation in different tissues.

Figure 4B shows that *OePPOs* also exhibit differential expression patterns in fruits, new leaves, and old leaves. Based on the clustering pattern in the figure, it can be observed that *OePPO-5* and *OePPO-8* are highly expressed in both fruits and new leaves, *OePPO-9* is mainly expressed in fruits, and *OePPO-3*, *4*, *7*, *10*, *11*, *12*, *14*, *15*, and *OePPO-16* are mainly expressed in new leaves. Similarly, the genes predominantly expressed in each tissue are not exclusively classified into Group 1 or Group 2 but are evenly distributed between the two groups. In fruits, *OePPO-5* and *OePPO-9* belong to Group 1 and Class III, while *OePPO-8* belongs to Group 2 and Class IV. In new leaves, *OePPO-3*, *4*, *7*, and *OePPO-15* belong to Group 1 and Class III, while *OePPO-10*, *11*, *12*, *14*, and *OePPO-16* belong to Group 2 and Class IV. Genes from both Group 1 and Group 2 are uniformly expressed in each tissue.

### 3.6. Browning Degree and PPO Activity Assay of Three Olive Varieties

The main economic value of the olive plant comes from olive oil production, meaning its economic value is primarily in its fruits. However, olive fruits have a soft texture and are susceptible to squeezing and collisions during harvesting, transportation, and processing, leading to mechanical damage and browning.

**Figure 5:**
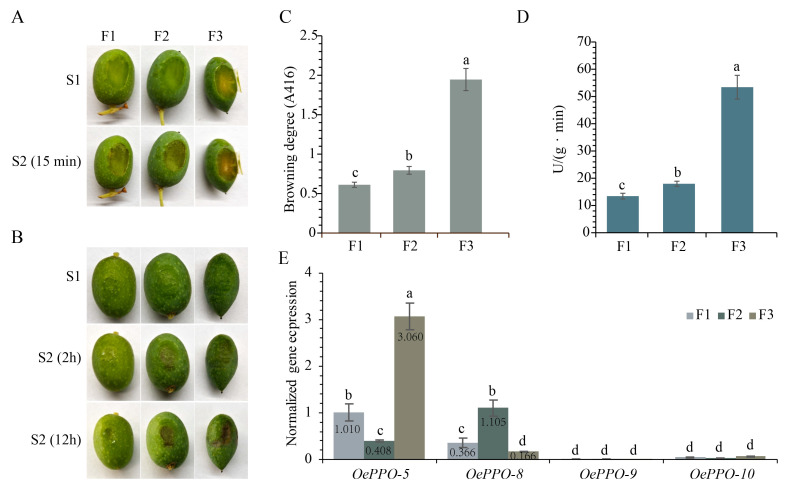
(**A**) Photographs of three fruits from each olive variety, after 0 and 15 mins of cutting; (**B**) photographs taken at 0, 2, and 12 h after induced mechanical pressure; (**C**) browning degree of the three fruits; (**D**) enzyme activity of the three fruits; and (**E**) expression of the four OePPOs (Different lowercase letters such as a, b, c, d indicate significant differences, *p* < 0.05).

In this study, three dwarf and high-density olive varieties were used as materials to investigate the differences in browning when subjected to mechanical damage (cutting and squeezing, Figure 5A,B). It was found that the Koroneiki (F3) exhibited the fastest degree of browning, followed by Arbosana (F2) and Arbequina (F1). The results of the browning degree (Figure 5C) also showed that F3 had the highest degree of browning, followed by F2 and F1.

PPOs play a crucial role in enzymatic browning, and we investigated the PPO enzyme activity in the three olive fruits. The results showed that F3 had the highest PPO activity, followed by F2 and F1 (Figure 5D). The trend of PPO activity among the three varieties was consistent with the trend of browning degree, indicating that the degree of browning increases with enhanced enzyme activity. This confirmed that the PPOs in olives are the key enzymes involved in enzymatic browning. These findings indicate that the rate of browning in olive fruit after mechanical damage is positively correlated with its PPO activity.

Subsequently, to further investigate which genes in the OePPO family are associated with enzymatic browning, we selected four genes, *OePPO-5*, *OePPO-8*, *OePPO-9*, and *OePPO-10*, based on their expression data in the fruit (Table S6). We performed RT-PCR with GAPDH2 as the internal reference gene (primer sequences in Table S6) and compared the expression levels to *OePPO-5* of F1 expression as the control. The results (Figure 5E) showed that *OePPO-5* and *OePPO-8* were primarily expressed, suggesting that *OePPO-5* and *OePPO-8* may play key roles in the browning process of olive fruit.

Of the three olive varieties, F3 showed a significantly higher browning degree and higher enzyme activity compared to F2 and F1. Additionally, the expression level of *OePPO-5* in F3 was significantly higher than in the other varieties or other genes. The correlation between the expression level of *OePPO-5* in F3 and the browning rate and PPO activity after mechanical damage strongly suggests that *OePPO-5* plays a key role in mediating the browning process in olive fruit.

## 4. Discussion

Through the local blast and tyrosinase domain structure, we identified 18 candidate *PPO* genes in the entire genome of the olive plant. Tran and Taylor [31] researched the number of *PPO* genes in 25 species belonging to five taxa (Chlorophytes, Bryophytes, Monocotyledonous Anthophytes, and Dicotyledonous Anthophytes). Among them, *Physcomitrella patens* has the highest number of *PPO* genes, with 13 *PPO* genes identified. *Arabidopsis thaliana* does not contain any *PPO* genes [20], and it only has laccases, which have functions similar to PPO. For example, the oxidative polymerization of flavonoids in the seed coat of Arabidopsis is catalyzed not by PPO but by laccase [51]. The loss of the PPO lineage in *Arabidopsis thaliana* indicates that the gene repertoire of certain species is mainly a result of independent bursts of gene duplication [51]. Similarly, the occurrence of tandem and segmental events in olives indicates that the formation of the olive gene repertoire also includes independent bursts of gene duplication. We identified 18 *PPO* genes in olives, which is much higher in number compared to most plants, suggesting that the *PPO* gene family plays a crucial role in the growth, development, and metabolism of olives. Olives are rich in characteristic metabolites such as oleuropein and hydroxytyrosol, and PPOs are believed to be involved in the synthesis of these related metabolites [28,29]. The higher number of *PPO* genes in olives compared to many other species is likely associated with the synthesis of its characteristic metabolites.

Plant PPOs are classified into two branches: one with diphenolase activity, known as COs, and the other with monophenolase activity, known as TYRs. Our research results also divided OePPO-1 to OePPO-18 into two groups, similar to the classification based on enzymatic activities. Group 1, mainly includes COs from dandelions, grapes, and apples [14]. OePPO-1 to OePPO-7, and OePPO-9, 15, and 18 cluster with them in this group, suggesting that these OePPOs may possess only diphenolase activity, similar to the COs found in the mentioned plants. Group 2, mainly includes TYRs from dandelions, grapes, and apples [14]. OePPO-8,10-14, 16, and 17, cluster with them in this group, suggesting that these OePPOs may possess both monophenolase and diphenolase activities, similar to the TYRs found in the mentioned plants. As a characteristic metabolite of olives, the synthesis of oleuropein mainly involves substrates such as tyrosine, tyramine, and tyrosol, which are catalyzed to form intermediate products such as DOPA, dopamine, and hydroxytyrosol [29]. This process requires the involvement of TYRs with monophenolase activity. Our research results suggest that *OePPO-8*, *10-14*, *16*, and *17*, may be the key genes involved in mediating oleuropein synthesis. Moreover, considering the gene expression data in fruits (Table S6), the highest expression of *OePPO-8* in fruits implies its importance as the main effector gene in this process.

In plants, different cultivars of the same species may exhibit varying degrees of browning when subjected to the same mechanical damage. This phenomenon is also observed in olives, as previously reported [52]. In this study, we measured the browning degree and PPO activity of three dwarf and densely planted olive cultivars. We observed that the degree of browning increases with enhanced enzyme activity. These observations indicate that there is a positive correlation between the browning degree of the fruit upon mechanical damage and PPO activity, suggesting that PPOs play a role in the enzymatic browning process of the olive fruit. Then, to identify the key genes involved in the browning process, we conducted an analysis of the expression levels of the *OePPO* gene family. Based on the gene expression data in Table S6, we found that *OePPO-5*, *OePPO-8*, *OePPO-9*, and *OePPO-10* are expressed in the fruit. Among them, OePPO-8 and OePPO-10 cluster together in Group 2, suggesting that they have monophenolase activity as TYRs and are involved in the synthesis of characteristic metabolites in olives, such as oleuropein and hydroxytyrosol. Therefore, it is likely that *OePPO-5* and *OePPO-9* are more closely related to the browning process in olive fruit. To further investigate, we performed RT-PCR experiments on the four genes expressed in the fruit. The RT-PCR results (Figure 5E) showed that among the four genes, *OePPO-5* and *OePPO-8* were primarily expressed, indicating that *OePPO-5* and *OePPO-8* are likely to be key genes involved in the browning process of olive fruit. It is worth noting that the expression level of *OePPO-5* in the F3 variety is significantly higher than in other varieties or other genes. Additionally, among the three olive varieties, the F3 fruit exhibits a significantly higher browning degree and enzymatic activity compared to F2 and F1. However, the situation was different for OePPO-8, where F2 showed the highest expression level. The differential expression of OePPO-5 and OePPO-8 could be attributed to functional differentiation. OePPO-5 only exhibited diphenolase activity, which is directly related to browning, and its expression in the fastest browning variety, F3, was significantly dominant. Therefore, we considered OePPO-5 as the main effector gene. Although the expression of OePPO-8 did not follow the trend of being highest in F3, followed by F2 and F1, we speculated that OePPO-8 was more likely to be the main effector gene involved in specialized metabolite synthesis, considering its dual mono-/diphenolase activity and the fact that PPOs with monophenolase activity are required in the synthesis of specialized metabolites such as hydroxytyrosol and oleuropein [28,29]. Furthermore, even when considering the diphenolase activity of OePPO-8, the total expression levels of both OePPO-5 and OePPO-8 aligned exactly with the total enzyme activity data, with F3 showing the highest expression, followed by F2, and F1 having the lowest expression. Based on the above, we believed that *OePPO-5* is the main effector gene involved in the enzymatic browning of olive fruit.

## 5. Conclusions

In conclusion, a total of 18 *OePPO* genes were identified in the olive genome. The OePPOs were classified into two groups, with OePPOs from Group 1 showing only diphenolase activity (COs) and OePPOs from Group 2 exhibiting both mono-/diphenolase activities (TYRs). The four most conserved motifs, motifs 5, 6, 9, and 12, are believed to be associated with differentiating COs and TYRs. Different olive fruit varieties were analyzed for browning degree and PPO activity. The results revealed a positive correlation between browning degree and PPO activity, indicating that PPO plays a crucial role in the enzymatic browning process of olive fruit. Furthermore, based on the expression levels, OePPO-5, which exhibits only diphenolase activity, was predicted to play a critical role in the browning process. Additionally, OePPO-8, which exhibits monophenolase activity, was predicted to be involved in the synthesis of specialized metabolites such as hydroxytyrosol and oleuropein. These findings provide a basis for further functional studies on the *OePPO* genes and to elucidate the mechanism of enzymatic browning in olive fruit, which could contribute to a reduction in the browning sensitivity of olive fruits in the future.

## Figures and Tables

**Table 1:** Selection pressure.

Gene Pair		Ka	Ks	Ka/Ks
OePPO-18	OePPO-5	0.36	2.35	0.15
OePPO-11	OePPO-10	0.12	0.29	0.42
OePPO-18	OePPO-9	0.28	0.9	0.32
OePPO-12	OePPO-13	0.08	0.17	0.46
OePPO-9	OePPO-7	0.29	1.83	0.16
OePPO-10	OePPO-14	0.11	0.19	0.58
OePPO-3	OePPO-6	0.37	1.82	0.2

## Data Availability

Data are contained within the article or Supplementary Material.

## Supplementary Materials

The following supporting information can be downloaded at: https://www.mdpi.com/article/10.3390/antiox12091661/s1, Table S1: PPO members in olive and their physicochemical properties; Table S2: GenBank accession of Plant PPOs; Table S3: GenBank accession numbers of plant PPOs; Table S4: Motif sequences; Table S5: FPKM values in six different tissues; Table S6: FPKM values in three different tissues; and Table S7: Oligonucleotides used for RT-qPCR.
