# Supplementary material for: Genome-Wide Analysis of the Polyphenol Oxidase Gene Family in Olea europaea Provides Insights into the Mechanism of Enzymatic Browning in Olive Fruit
Source: Antioxidants (Basel). 2023 Aug 23;12(9):1661. doi: 10.3390/antiox12091661 (PMC10525835; doi:10.3390/antiox12091661)
Supplement: Supplementary file 1 [file antioxidants-12-01661-s001.zip › antioxidants-2544322-supplementary.pdf]

Table S1. PPO members in olive and their physicochemical properties

Database and accession numbers		Name	Protein size (aa)	Tyrosinase domain		PPO1_DWL domain		PPO1_KFDV domain		MW (KDa)	pI	TMHs
				pfam Accsssion	Position (aa)	pfam Accsssion	Position (aa)	pfam Accsssion	Position (aa)			
Olive genome database	EVM0041467	*OePPO-1*	581	pfam00264	166-375	pfam12142	384-434	pfam12143	457-573	65.03	6.15	0
	EVM0050366	*OePPO-2*	581	pfam00264	166-375	pfam12142	384-434	pfam12143	457-573	65.03	6.15	0
	EVM0028836	*OePPO-3*	582	pfam00264	167-376	pfam12142	385-435	pfam12143	458-574	65.16	7.6	0
	EVM0049750	*OePPO-4*	582	pfam00264	160-372	pfam12142	379-429	pfam12143	455-571	65.96	5.37	0
	EVM0025110	*OePPO-5*	573	pfam00264	157-365	pfam12142	372-422	pfam12143	445-570	64.4	6.61	0
	EVM0038700	*OePPO-6*	561	pfam00264	148-355	pfam12142	363-413	pfam12143	434-559	63.86	7.29	0
	EVM0020212	*OePPO-7*	597	pfam00264	186-392	pfam12142	399-447	pfam12143	471-588	66.94	7.23	0
	EVM0039718	*OePPO-8*	557	pfam00264	182-391	pfam12142	397-448	cl15965	476-552	62.88	7.26	0
	EVM0034699	*OePPO-9*	567	pfam00264	152-356	pfam12142	369-418	pfam12143	441-546	64.31	6.04	0
	EVM0023965	*OePPO-10*	584	pfam00264	174-387	pfam12142	392-443	pfam12143	463-582	66.12	6.04	0
	EVM0008012	*OePPO-11*	568	pfam00264	157-370	pfam12142	375-426	pfam12143	446-566	63.96	6.6	0
	EVM0018385	*OePPO-12*	568	pfam00264	157-370	pfam12142	375-426	pfam12143	446-566	63.8	6.47	0
	EVM0021447	*OePPO-13*	575	pfam00264	164-377	pfam12142	382-433	pfam12143	453-573	64.81	6.22	0
	EVM0052187	*OePPO-14*	528	pfam00264	165-348	pfam12142	353-396	pfam12143	407-526	59.15	6.11	0
	EVM0005988	*OePPO-15*	326	cl02830	157-320	-		-		36.36	8.62	0
	EVM0054558	*OePPO-16*	365	cl02830	8-167	pfam12142	172-223	pfam12143	243-363	41.6	5.23	0
	EVM0022924	*OePPO-17*	306	pfam00264	52-225	pfam12142	230-279	-		35.84	6.29	0
	EVM0008425	*OePPO-18*	444	cl02830	141-284	cl13563	286-322	cl15965	346-435	49.34	7.11	0

Table S2. GenBank accession of Plant PPOs

Species	GenBank accession	Rename
Populus trichocarpa	XP_002316852.1	PtPPO-1
	XP_006370115.2	PtPPO-2
	XP_024445363.1	PtPPO-3
	XP_006370127.2	PtPPO-4
	XP_006370118.1	PtPPO-5
	XP_006370120.2	PtPPO-6
	XP_024448244.1	PtPPO-7
	XP_006370125.2	PtPPO-8
	XP_024448245.1	PtPPO-9
	XP_024464832.1	PtPPO-10
	XP_002316632.1	PtPPO-11
	XP_006370128.1	PtPPO-12
	XP_002305680.2	PtPPO-13
Physcomitrium patens	XP_024403506.1	PpPPO-1
	XP_024361473.1	PpPPO-2
	XP_024363392.1	PpPPO-3
	XP_024384986.1	PpPPO-4
	XP_024361667.1	PpPPO-5
	XP_024361666.1	PpPPO-6
	XP_024361665.1	PpPPO-7
	XP_024396277.1	PpPPO-8
	XP_024397287.1	PpPPO-9
	XP_024401964.1	PpPPO-10
	XP_024358854.1	PpPPO-11
	XP_024403288.1	PpPPO-12
	XP_024383156.1	PpPPO-13

Species	GenBank accession	Rename
Solanum lycopersicum	XP_004232833.3	SiPPO-1
	NP_001318059.1	SiPPO-2
	XP_004246041.1	SiPPO-3
	NP_001318057.1	SiPPO-4
	NP_001296326.1	SiPPO-5
	NP_001334885.1	SiPPO-6
	XP_004246029.3	SiPPO-7
	XP_004246037.1	SiPPO-8
Selaginella moellendorffii	XP_024519810.1	SmPPO-1
	XP_024540745.1	SmPPO-2
	XP_002974314.2	SmPPO-3
	XP_024537619.1	SmPPO-4
	XP_024541491.1	SmPPO-5
	XP_024541385.1	SmPPO-6
	XP_024537620.1	SmPPO-7
	XP_024531515.1	SmPPO-8
	XP_024529963.1	SmPPO-9
	XP_002982245.2	SmPPO-10
	XP_024541271.1	SmPPO-11
Sorghum bicolor	XP_002446944.1	SbPPO-1
	XP_002446945.1	SbPPO-2
	XP_002458623.1	SbPPO-3
	XP_021311316.1	SbPPO-4
	XP_002438681.1	SbPPO-5
	XP_002443986.1	SbPPO-6
	XP_002443987.1	SbPPO-7
	XP_002445175.1	SbPPO-8

Table S3. GenBank accession numbers of plant PPOs. The tyrosinase domains of the listed plant PPOs were phylogenetically analyzed.

Enzyme	Organism	Accession
AmAS1	*Antirrhinum majus*	BAB20048.1
CgAUs1	*Coreopsis grandiflora*	AHN09736.1
Ibco	*lpomoea batatas* 'Yushu-303'	AAW78869.1
JrTYR	*Juglans regia* 'Chandler'	ACN86310.1
LtLH	*Larrea tridentata*	AAQ67412.1
MdPPO1	*Malus domestica* 'Golden Delicious'	SJK83184.1
ToPPO-1	*Tarraxacum officinale*	ABX09994.1
ToPPO-2	*Tarraxacum officinale*	CAQ76694.1
ToPPO-3	*Tarraxacum officinale*	CBZ41490.1
ToPPO-4	*larraxacum officinale*	CBZ41491.1
ToPPO-5	*Tarraxacum officinale*	CBZ41492.1
ToPPO-6	*Tarraxacum officinale*	CCA94610.1
ToPPO-7	*Tarraxacum officinale*	CCD61123.1
ToPPO-8	*Tarraxacum officinale*	CCD61124.1
ToPPO-9	*Tarraxacum officinale*	CCD61125.1
ToPPO-10	*Tarraxacum officinale*	CCE45701.1
ToPPO-11	*Tarraxacum officinale*	CCD61126.1
VvPPOcs-3	*Vitis vinifera* 'Cabernet Sauvignon'	ALP46091.1
VvPPOg	*Vitis vinifera* 'Grenache'	AAB41022.1

Table S4. Motif sequences

Motif ID	MotifSeq	Length
Motif 1	FMQQANVHCAYCBGAYDQVGYNNLDLZVHNSWLFFPFHRWYLYFFERILG	50
Motif 2	EDMGNFYSAGRDPIFYAHHANVDRMWTIWRB	31
Motif 3	EILVJEGIEVDTTKYVKFBVFINDEDDNASELDNAEYAGSFAQVPHKHKM	50
Motif 4	KGKBITDPDWLNAYFJFYBENAKLVRVKIQDCLDTTKLGYTYEDVP	46
Motif 5	NLIDDPTFALPFWNWDNPKGMTIPPMFBSS	30
Motif 6	NNLTIMYSZMIRQSKTPTDFFGNPYRAGD	29
Motif 7	AAHTVDSEYIAKYEEAIQRMKNLDPTDPR	29
Motif 8	KTNIRLRJTELLEEJDVEDDDTVLVTJVPRSN	32
Motif 9	PGAGSIERGSHTAVHSWVGT	20
Motif 10	QGKJDRRBVLLGLGGLYGASNLVNN	25
Motif 11	APDAAQVFPVTLDKPIRVIVPRPKKSKSG	29
Motif 12	SPLYDAKRBPBHLPP	15
Motif 13	MASLQASATVTSCIPYSSSFS	21
Motif 14	DVTIGGIKIVF	11
Motif 15	GDALDINCCPP	11

Table S5. FPKM values in six different tissues

	FT-1	FT-2	FT-3	FT-4	FR-1	FR-2	FR-3	FR-4	LF-1	LF-2	LF-3	LF-4
OePPO-1	0	0	0	0	0.2346	0.3096	0.3780	0.3478	0	0.0193	0	0
OePPO-2	0	0	0	0	0.0065	0.0035	0.0053	0.0203	0	0	0	0
OePPO-3	0	0	0.0179	0.0179	92.9306	96.3090	95.9223	93.8868	0.0188	0.0190	0.0336	0.0000
OePPO-4	0	0.0364	0	0	0.3498	0.4942	0.2808	0.3505	0	0	0	0.0352
OePPO-5	7.0024	6.4788	4.6853	4.4169	9.3757	9.4857	9.0732	9.2291	3.3874	3.2790	7.1394	8.0799
OePPO-6	0	0	0	0	0	0	0	0	0	0	0	0
OePPO-7	0	0	0	0	0	0	0	0	0	0	0	0
OePPO-8	0.3005	0.2987	2.5395	1.5876	2.5337	2.4976	2.9271	2.2025	0.4566	0.4373	0.5599	0.7710
OePPO-9	0.0000	0.0449	0.0699	0.0000	126.9102	126.3880	126.3065	125.9410	0	0	0	0
OePPO-10	7.1586	6.7827	2.4614	2.4469	35.1531	35.2408	32.7337	33.8921	0.5434	0.2826	0.5019	0.4723
OePPO-11	0	0	0	0	0.3520	0.3779	0.2105	0.2497	0	0	0	0
OePPO-12	0	0	0	0	0.2107	0.2116	0.1054	0.0596	0	0	0	0
OePPO-13	0	0	0.0178	0	0.1223	0.0970	0.0717	0.0601	0	0	0	0
OePPO-14	0	0	0.0000	0	9.7492	10.7054	10.3009	10.3176	0.3400	0.1796	0.2857	0.2046
OePPO-15	0	0	0.0697	0.0693	127.7499	123.1864	132.9123	125.1700	0.0885	0.0693	0	0
OePPO-16	0	0	0	0	0.1498	0.1855	0.1676	0.2277	0	0	0	0
OePPO-17	0	0	0	0	0	0	0	0	0	0	0	0
OePPO-18	0	0	0	0	0	0	0	0	0	0	0	0

Table S5. Continue

	MM-1	MM-2	MM-3	MM-4	RT-1	RT-2	RT-3	RT-4	SM-1	SM-2	SM-3	SM-4
OePPO-1	0	0	0	0	0.0549	0.0365	0.0595	0.0944	0	0	0	0
OePPO-2	0	0	0	0	0.0549	0.0365	0.0595	0.0944	0	0	0	0
OePPO-3	1.4070	1.3184	0.1507	0.1586	1.5975	1.5694	0.2909	0.0797	0.0890	0.0207	0.0194	0.0707
OePPO-4	14.1352	14.0042	13.4923	13.3573	0	0	0.0489	0	0.3297	0.6026	0.2029	0.1833
OePPO-5	36.5332	35.5518	36.4884	37.5374	1.9974	2.1519	1.0176	0.8915	2.0550	1.9088	1.5578	1.5128
OePPO-6	0	0	0	0	0.0381	0	2.1398	1.3806	0	0	0	0
OePPO-7	0	0	0	0	0	0	0	0	0	0	0	0
OePPO-8	11.9123	11.9901	6.6384	6.8451	1.5741	1.7445	1.7740	2.5187	3.0527	3.0011	2.0714	2.3413
OePPO-9	0.0532	0.1768	0	0	5.2237	5.8464	3.4839	2.6632	0.0460	0.0668	0.0410	0
OePPO-10	15.9928	15.8605	20.6007	19.7054	18.9778	20.0102	22.2327	23.4916	2.4703	2.1908	3.7220	4.1480
OePPO-11	0.6028	0.9154	0.5326	0.2201	0.0325	0.0239	0.0244	0.0624	0	0	0	0
OePPO-12	0.0235	0.0505	0.0033	0.0599	0.2183	0.2124	0.0478	0.0593	0	0	0	0
OePPO-13	0	0	0.0029	0	13.0807	13.3106	12.4222	14.1134	0	0	0.0420	0
OePPO-14	4.8438	5.0187	3.3721	3.2785	0.0622	0	0.0736	0	0	0	0.1603	0
OePPO-15	3.4142	2.9492	0.3651	0.5249	0.1791	0.2233	0	0	0.0994	0	0	0
OePPO-16	0.1390	0.4019	0.0000	0.2183	0.0544	0.0604	0.2989	0.0625	0	0	0	0
OePPO-17	0	0	0	0	0	0	0	0	0	0	0	0
OePPO-18	0	0	0	0	0	0	0	0	0	0	0	0

Table S6. FPKM values in three different tissues

	F1	F2	F3	NL1	NL2	NL3	OL1	OL2	OL3
OePPO-1	0	0	0	0	0	0	0	0	0
OePPO-2	0	0	0	0	0	0	0	0	0
OePPO-3	0	0	0	3.1295	3.1454	3.2039	0	0	0
OePPO-4	0	0	0	4.2718	4.2354	4.4119	0	0	0
OePPO-5	14.4240	13.9722	13.6553	11.6473	11.5027	10.6635	2.8938	2.4767	2.7971
OePPO-6	0	0	0	0	0	0	0	0	0
OePPO-7	0	0	0	0	0.0194	0	0	0	0
OePPO-8	4.9150	4.3320	3.8717	8.2118	7.9320	7.5015	0.9590	0.3708	1.1750
OePPO-9	1.7827	2.0073	2.0801	0.0000	0.0476	0	0	0	0
OePPO-10	1.5492	1.5037	1.8773	13.8004	12.9862	13.2802	0.9525	0.8729	1.4718
OePPO-11	0	0	0	1.1693	0.9726	0.5712	0	0	0
OePPO-12	0	0	0	0	0.0218	0	0	0	0
OePPO-13	0	0	0	0	0	0	0	0	0
OePPO-14	0.0435	0	0	4.1235	3.8069	3.9304	0.0846	0.3342	0.2941
OePPO-15	0	0	0	7.1616	7.7683	7.7439	0	0	0
OePPO-16	0	0	0	0.0311	0.0706	0.0812	0	0	0
OePPO-17	0	0	0	0	0	0	0	0	0
OePPO-18	0	0	0	0	0	0	0	0	0

Table S7. Oligonucleotides used for RT-qPCR.

Name	Sequence (5'-3')
qOePPO-5F	TGACGGGAAGAGTCCTTTGC
qOePPO-5R	AATGGATCGCAGTGTGCGTA
qOePPO-8F	GTTAGGAGGACTGTACGGCG
qOePPO-8R	GTATTTCTCCGGTGGGTGGG
qOePPO-9F	TGACATAAGGCACAACTGCGA
qOePPO-9R	TTCGCTTCCCTGAACTGGTG
qOePPO-10F	TCAACGACGATGCAACGGTA
qOePPO-10R	GAGGCACTGATCTTGGGGAC
qGAPDH2-F	CCTTCCGTGTGCCTACTGTT
qGAPDH2-R	GATGGCTGCCTTGATTTCAT
